# Development and Validation of a Prediction Model for Positive Margins in Breast-Conserving Surgery

**DOI:** 10.3389/fonc.2022.875665

**Published:** 2022-05-12

**Authors:** Rong Zhao, Jun Xing, Jinnan Gao

**Affiliations:** Department of Breast Surgery, Shanxi Bethune Hospital, Shanxi Academy of Medical Sciences, Tongji Shanxi Hospital, Third Hospital of Shanxi Medical University, Taiyuan, China

**Keywords:** breast cancer, breast-conserving surgery, excision margin, nomogram, decision making

## Abstract

**Background:**

The chances of second surgery due to positive margins in patients receiving breast-conversing surgery (BCS) were about 20-40%. This study aims to develop and validate a nomogram to predict the status of breast-conserving margins.

**Methods:**

The database identified patients with core needle biopsy-proven ductal carcinoma *in situ* (DCIS) or invasive breast carcinoma who underwent BCS in Shanxi Bethune Hospital between January 1, 2015 and December 31, 2021 (n = 573). The patients were divided into two models: (1) The first model consists of 398 patients who underwent BCS between 2015 and 2019; (2) The validation model consists of 175 patients who underwent BCS between 2020 and 2021. The development of the nomogram was based on the findings of multivariate logistic regression analysis. Discrimination was assessed by computing the C-index. The Hosmer-Lemeshow goodness-of-fit test was used to validate the calibration performance.

**Results:**

The final multivariate regression model was developed as a nomogram, including blood flow signals (OR = 2.88, p = 0.001), grade (OR = 2.46, p = 0.002), microcalcifications (OR = 2.39, p = 0.003), tumor size in ultrasound (OR = 2.12, p = 0.011) and cerbB-2 status (OR = 1.99, p = 0.042). C-indices were calculated of 0.71 (95% CI: 0.64-0.78) and 0.68 (95% CI: 0.59-0.78) for the modeling and the validation group, respectively. The calibration of the model was considered adequate in the validation group (p > 0.05).

**Conclusion:**

We developed a nomogram that enables the estimation of the preoperative risk of positive BCS margins. Our nomogram provides a valuable tool for identifying high-risk patients who might have to undergo a wider excision.

## Introduction

Women with early-stage breast cancer are typically treated with breast-conserving surgery (BCS). In BCS, clean surgical margins prevent recurrence (1). However, according to the literature, a positive margin status after BCS increases the risk of reoperation by 20-40% (2). It is very well established that secondary operations are associated with financial, health, and psychological implications (3–6). The intraoperative margin can be evaluated using various techniques, such as gross analysis, fluorescent techniques, cytology, frozen section procedure, high-frequency ultrasound, and radiofrequency spectroscopy (7, 8). The extent of its use depends on the preferences of the treating center. According to evidence, the frozen section technique is highly accurate for evaluating the intraoperative margin of BCS (9) and reducing reoperation rates (3, 10). However, its complexity, time-consuming nature, and heavy workload limit its universal acceptance (7, 10).

The literature recommends a wider excision for patients with high-risk positive margins to reduce re-excision during or after the surgery. The recent evidence indicates that factors such as age, tumor type, tumor size, multifocal disease, tumor grade, extensive intraductal component (EIC), lymphovascular invasion (LVI), microcalcifications on mammography and lymph node stage are potentially associated with margin status (11, 12). However, information about some factors can only be obtained after BCS on paraffin-embedded specimens. For example, accurate axillary nodal stage relays on pathological methods and is only obtained after surgery. Several nomograms exist that can predict the breast-conserving margins using preoperative massage (13–18), however, most nomograms are based on the prior definition of “positive margin” that was revised in 2014 (19). Therefore, this study aims to identify the preoperative predictors of margin status to develop a nomogram for predicting operative margins. Hence, the outcome of this study will facilitate surgeon and patient decision-making on wider excision ahead of BCS.

## Patients and Methods

### Patients

The institutional review board of Shanxi Bethune Hospital approved the study protocols. Data were obtained from the Shanxi Bethune Hospital from January 1, 2015 and December 31, 2021, which included patients with ductal carcinoma *in situ* (DCIS) or invasive breast cancer with confirmed malignancy by core needle biopsy and who underwent BCS (n= 616). Out of 616, 43 patients were excluded because of neoadjuvant therapy or the absence of key information about margin status, which cannot be determined from the database. Data from the remaining 573 patients were analyzed, out of which, 398 patients who received BCS (2015 – 2019) were used to develop a nomogram. For model validation, data from 175 patients who underwent BCS (2020- 2021) were used. The outcome variable was the status of permanent margin. The number of outcomes in our modeling cohort was 74, and 5 predictors were selected. The sample size was adequate based on the events per variable principle (20).

### Clinicopathological Information

In addition to the information available in databases (age, BMI), we gathered information about palpability, tumor location, imaging features, and margin status from electronic medical records. Imaging features were obtained from ultrasonography, mammography, and breast magnetic resonance imaging (MRI) reports routinely performed before a breast-conserving operation. Ultrasound reports were used to determine the maximum diameter of tumors and features of malignancy such as spicules, crab signs, and blood flow. Diameter of tumors was treated as a categorical variable and the cutoff value was 2cm. Mammography mainly detects microcalcifications, asymmetric density, and distorted structure. Since MRI is a highly sensitive process, it primarily evaluated multiple lesions. A core needle biopsy was performed on all patients and examined by immunohistochemistry, reporting estrogen receptor (ER) status, progesterone receptor (PR) status, Her2/neu receptor status, histological grade, lymphovascular invasion (LVI) and Ki67. ER and PR positivity was defined as >1% positive tumor cells with nuclear staining. HER2/neu status was positive in the case of Her-2/neu 3+ or Her-2/neu 2+ with positive fluorescence *in situ* hybridization (FISH). Ki67 was treated as a categorical variable and the cutoff value was 30% (21).

### Surgical Procedure

In this study, all breast lumps were evaluated by pathological examination of the intraoperative frozen section. After receiving the tissue in the laboratory, it was immediately “annotated” to represent the *in vivo* position correctly. The sections for margin evaluation were taken perpendicularly to the inked surface. Microscopic measurements can be made to determine the distance of the carcinoma from the inked margin. The positive definition is in accordance with the current guideline (19, 22): on inked margins for invasive cancer and margins less than 2 mm for DCIS. The positive margins were re-excised and the new margin was analyzed by intraoperative frozen section analysis. For permanent results, all samples were paraffin-embedded and tested after BCS. Reoperation was recommended if the permanent margin was unclear (except for a positive frozen margin with re-excision). The outcome of our study was permanent positive margins and all the data were from the pathology reports.

### Statistical Analysis

The modeling and validation cohorts for missing values were initially performed before analysis. The proportion of missing data was less than 5% among the predictors. Multiple imputations were used for the missing data [predictive mean matching is embedded with the cases (k = 5 default)]. Univariate analyses were used to compare margin status in the modeling cohort. Fisher’s exact test was utilized for categorical variables. Variables with a p-value < 0.1 on the univariate test were included in multivariate logistic regression analysis. In addition, their clinical relevance and ability to improve model accuracy were considered. The C-index calculated discrimination in two cohorts. Calibration was assessed graphically by plotting the actual proportions against the predicted probabilities. The model’s overall fit was evaluated using the Hosmer-Lemeshow goodness-of-fit test. Statistical analyses were performed using IBM SPSS statistics version 26.0 software (IBM Corp., Armonk, USA) and the R software (version 4.0.0). Two-sided P-value < 0.05 was considered statistically significant. Our study followed the TRIPOD (transparent reporting of a multivariable prediction model for individual prognosis or diagnosis) statement (23).

## Results

Patients and tumor characteristics of the modeling and the validation group are listed in **Table 1**. A total of 119 (20.8%) patients had positive margins, 18.6% (74 of 398) in the modeling group, and 25.7% (45 of 175) in the validation group, respectively.

**Table 1:** Patient demographics and clinicopathologic characteristics in the study and validation cohort.

	Study cohort (n=398)	Validation cohort (n=175)
Period	2015.1-2019.12	2020.1-2021.12
Positive margins	74 (18.6%)	45 (25.7%)
Age, years (median, range)	51 (25-63)	55 (26-93)
BMI (median, range)	24.8 (17.0-38.6)	25.1 (16.8-35.3)
Tumor location		
Outer upper quadrant	156 (39.2%)	75 (42.9%)
Palpability	366 (92.0%)	154 (88.0%)
Multiple lesions	23 (5.8%)	15 (8.6%)
Ultrasonographic features		
Tumor size		
<= 2cm	236 (59.3%)	95 (54.3%)
>2cm	162 (40.7%)	80 (45.7%)
Spiculated margin	145 (36.4%)	67 (38.3%)
Crab sign	104 (26.1%)	57 (32.6%)
Increased blood flow	71 (17.8%)	44 (25.1%)
mammographic features		
Distorted structure	11 (2.8%)	7 (4.0%)
Asymmetric density	33 (8.3%)	10 (5.7%)
Microcalcifications	114 (28.6%)	58 (33.1%)
ER positive	297 (74.6%)	137 (78.3%)
PR positive	266 (66.8%)	111 (63.4%)
cerbB-2 positive	71 (17.8%)	37 (21.1%)
Ki-67		
Ki67 <= 30%	201 (50.5%)	96 (54.9%)
Ki67 > 30%	197 (49.5%)	79 (45.1%)
LVI	97 (24.4%)	28 (16.0%)
Grade		
1-2	230 (57.8%)	98 (56.0%)
3	168 (42.2%)	77 (44.0%)

BMI, Body Mass Index; ER, estrogen receptor; PR, progesterone receptor; LVI, lymphovascular invasion.

### Development of Nomogram

Predictors of the modeling group are listed in **Table 2**. The predictors for positive and negative margins with a p value < 0.1 include palpability (p = 0.060), tumor size on ultrasound (p = 0.018), increased blood flow (p = 0.001), microcalcifications (p = 0.002), cerbB-2 status (p = 0.053), and grade (p = 0.008). Later, the inclusion of palpability in the Logistic model was not considered necessary since it did not enhance the model’s accuracy.

**Table 2:** Comparison of variables with clear and positive resection margins in the study cohort.

	Positive margins (n=74)	Clear margins (n=324)	OR (95% CI)	P
Age (years)			1.11 (0.67-1.85)	0.675
< 50	35 (47.3%)	162 (50.0%)		
≥ 50	39 (52.7%)	162 (50.0%)		
Tumor location			1.00 (0.60-1.68)	1.000
Outer upper quadrant	29 (39.2%)	127 (39.2%)		
Other quadrant	45 (60.8%)	197 (60.8%)		
Palpability			0.47 (0.21-1.03)	0.060
no	10 (13.5%)	22 (6.8%)		
yes	64 (86.5%)	302 (93.2%)		
Multiple lesions			1.59 (0.61-4.19)	0.345
no	68 (91.9%)	307 (94.8%)		
yes	6 (8.1%)	17 (5.2%)		
Tumor size in ultrasound			0.51 (0.30-0.89)	0.018
≤ 2cm	53 (71.6%)	183 (56.5%)		
>2cm	21 (28.4%)	141 (43.5%)		
Spiculated margin			1.16 (0.69-1.94)	0.585
no	45 (60.8%)	208 (64.2%)		
yes	29 (39.2%)	116 (35.8%)		
Crab sign			1.06 (0.60-1.87)	0.846
no	54 (73.0%)	240 (74.1%)		
yes	20 (27.0%)	84 (25.9%)		
Increased blood flow			2.59 (1.45-4.63)	0.001
no	51 (68.9%)	276 (85.2%)		
yes	23 (31.1%)	48 (14.8%)		
Distorted structure			2.59 (0.74-9.08)	0.138
no	70 (94.6%)	317 (97.8%)		
yes	4 (5.4%)	7 (2.2%)		
Asymmetric density			1.20 (0.50-2.87)	0.687
no	67 (90.5%)	298 (92.0%)		
yes	7 (9.5%)	26 (8.0%)		
Microcalcifications			2.25 (1.33-3.80)	0.002
no	42 (56.8%)	242 (74.7%)		
yes	32 (43.2%)	82 (25.3%)		
ER status			1.02 (0.57-1.82)	0.948
positive	55 (74.3%)	242 (74.7%)		
negative	19 (25.7%)	82 (25.3%)		
PR status			0.96 (0.56-1.65)	0.882
positive	50 (67.6%)	216 (66.7%)		
negative	24 (32.4%)	108 (33.3%)		
cerbB-2 status			0.55 (0.30-1.01)	0.053
positive	19 (25.7%)	52 (16.0%)		
negative	55 (74.3%)	272 (84.0%)		
Ki67			1.34 (0.81-2.22)	0.261
≤ 30%	33 (44.6%)	168 (51.9%)		
> 30%	41 (55.4%)	156 (48.1%)		
LVI			1.09 (0.62-1.94)	0.760
no	54 (73.0%)	242 (74.7%)		
yes	20 (27.0%)	82 (25.3%)		
Grade			0.48 (0.28-0.83)	0.008
1-2	53 (71.6%)	177 (54.6%)		
3	21 (28.4%)	147 (45.4%)		

ER, estrogen receptor; PR, progesterone receptor; LVI, lymphovascular invasion.

**Figure 1:**
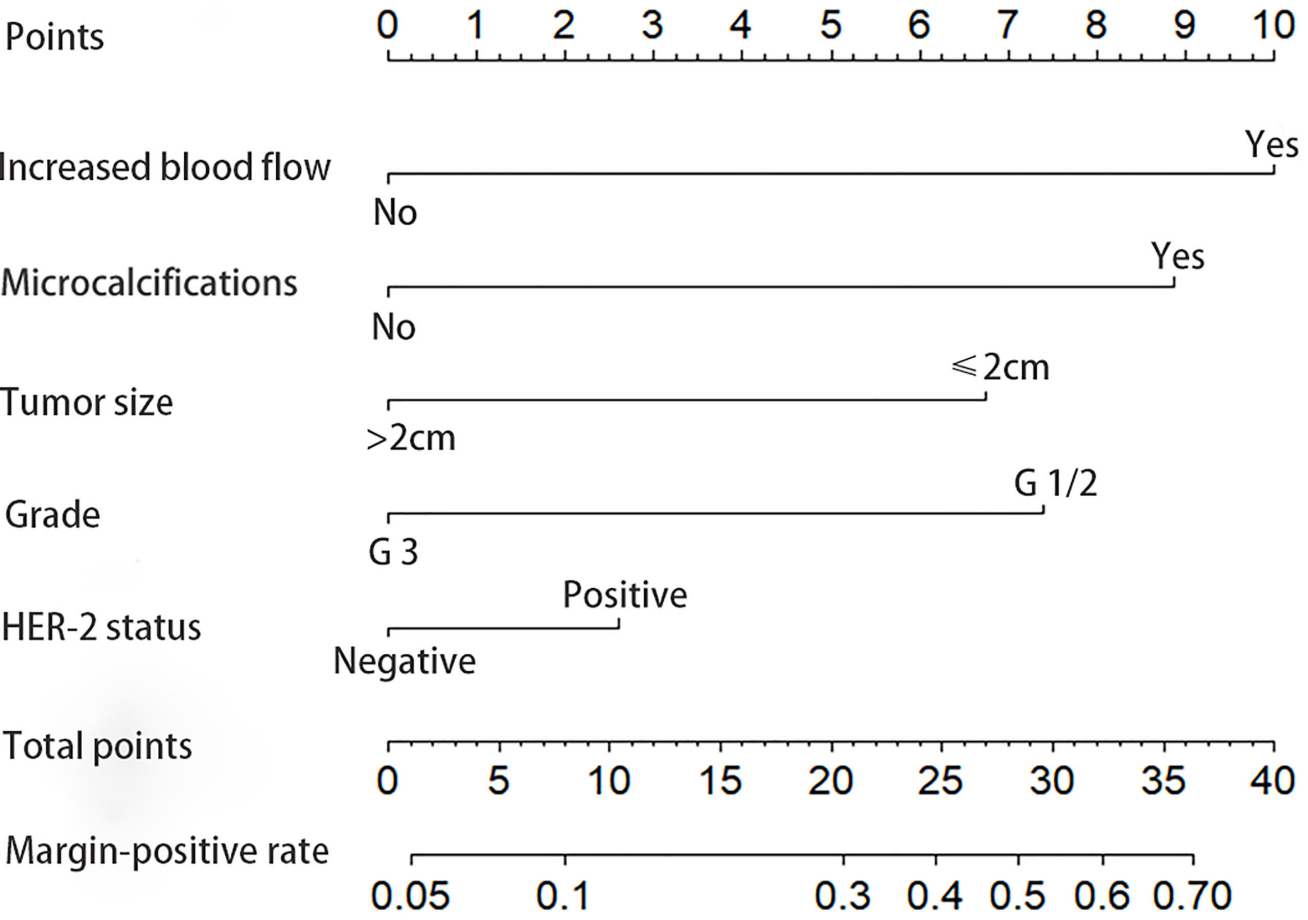
Nomogram for predicting positive resection margins in breast-conserving surgery.

The final model is presented in **Table 3**. The best discriminators between positive and negative margins were blood flow signals (OR = 2.88, p = 0.001), grade (OR = 2.46, p = 0.002), microcalcifications (OR = 2.39, p = 0.003), tumor size in ultrasound (OR = 2.12, p = 0.011) and cerbB-2 status (OR = 1.99, p = 0.042). A graphical nomogram was developed based on the results of logistic regression, **Figure 1**.

**Table 3:** Multivariate logistic regression model for positive resection margins in the modeling cohort.

Variables	OR	95%CI	P
Tumor size in ultrasound (≤2cm vs. >2cm)	2.12	1.19-3.80	0.011
Increased blood flow(yes vs. no)	2.88	1.55-5.35	0.001
Microcalcifications(yes vs. no)	2.39	1.37-4.17	0.003
cerbB-2 status(positive vs. negative)	1.99	1.02-3.85	0.042
Grade(G1/2 vs. G3)	2.46	1.37-4.44	0.002

### Evaluation of the Model

**Figure 2:**
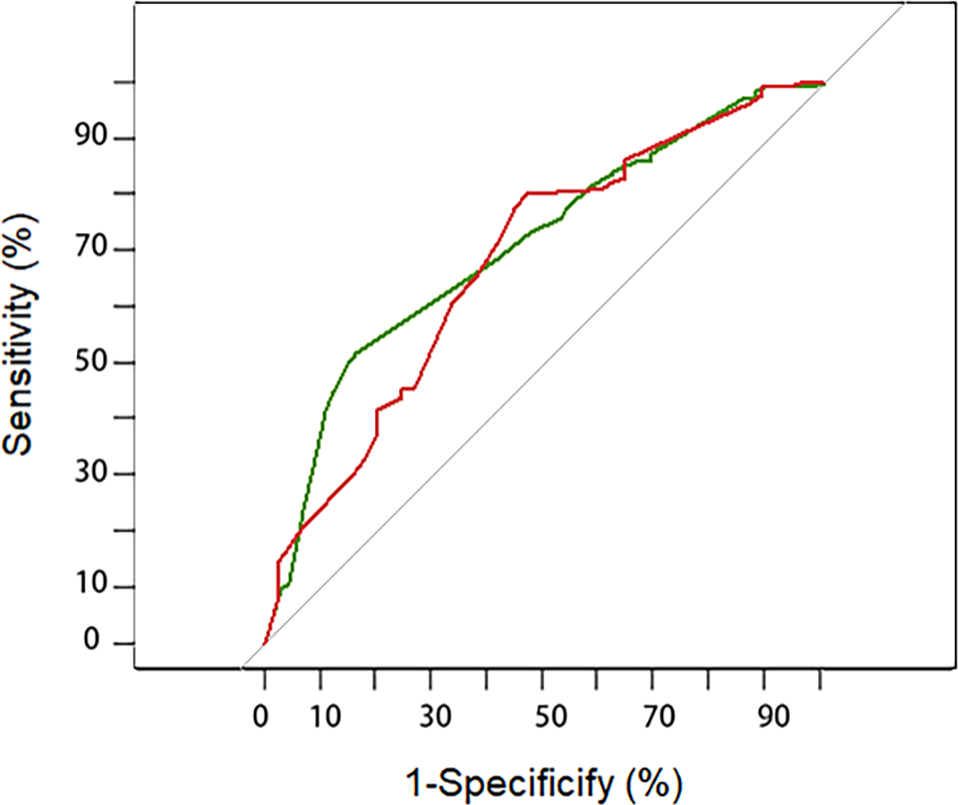
Receiver–operating characteristic curve for the prediction model in the development (green line) and validation cohort (red line). The AUC (area under the curve) indicated the discriminative power of the nomogram. The modeling group has a value of 0.71 (95%CI: 0.64-0.78) and the validation group has a value of 0.68 (95%CI: 0.59-0.78).

**Figure 3:**
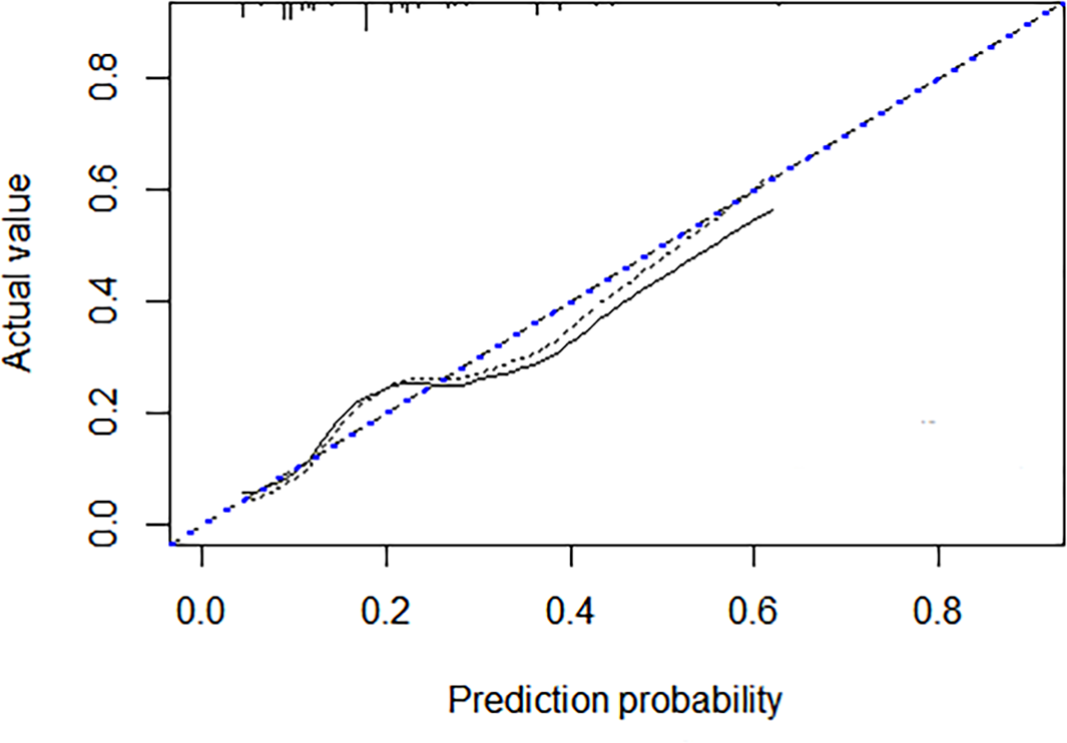
Calibration plot of the nomogram using the validation cohort.

A discrimination assessment was conducted for the modeling and validation group (**Figure 2**). The C-index was 0.71 (95% CI: 0.64-0.78) in the modeling group and 0.68 (95% CI: 0.59-0.78) in the validation group. Calibration was evaluated using an independent validation cohort and considered acceptable (Hosmer–Lemeshow goodness-of-fit, p = 0.058) (**Figure 3**).

### Instruction of the Model

The values were marked, read off, and individual scores were summed for each of the five predictors. The total score was marked on the axis at the bottom of the graph and the corresponding estimated probability of positive resection margins was read. Our model provides the user with a patient-tailored estimation of the preoperative risk of positive margins, stratified as low (< 10%), intermediate (10-30%), or high (> 30%) risk.

## Discussion

The low breast-conserving rate in China has been partially attributed to the psychological resistance of patients to have a second surgery (24). In China, many institutions assess intraoperative frozen margin as it is a reliable method of reducing the rate of reoperation. However, this method consumes a significant amount of time and workforce. In patients with positive frozen margins, re-excision is usually required intraoperatively, followed by waiting for the second margin. Omitting intraoperative assessment for patients with a low probability of having a positive frozen margin and recommending high-risk patients to receive oncoplastic surgery with wider margins may be a solution. Thus, the objective of our study is to create a model to identify patients at low and high risks with positive margins.

Previous studies have reported several models (13–18) (**Table 4**). However, most previous models were based on the definition of a positive margin proposed earlier than 2014. In 2014, the definition of “positive margin” for invasive cancer was updated to “no ink on tumor”. Recent evidence shows that the updated standard is associated with a low incidence of ipsilateral breast tumors recurrence (IBTR). Furthermore, it can decrease re-excision rates, improve cosmetic outcomes, and decrease health care costs (25). To ensure consistency of results we selected patients after 2015.

**Table 4:** Comparison with previous model predicting likelihood of positive margin in BCS patients.

Study	Population	Rate of positive margins	Variables in model	OR	95% CI	AUC	
						model	validation
Shin et al. (14)	Patients with invasive or *in situ* palpable and non-palpable breast cancer	14.6%(151/1034)	Microcalcifications on mammogram	1.57	1.04-2.39	0.82	0.85
			Breast density on mammogram				
			Type 2	1.59	0.53-4.81		
			Type 3	1.61	0.56-4.62		
			Type 4	4.52	1.57-12.95		
			>0.5 cm difference MRI e ultrasound	10.0	6.50-15.39		
			DCIS present on needle biopsy	1.58	1.01-2.45		
			Lobular component on needle biopsy	3.99	1.31-12.12		
Pleijhuis et al. (15)	Patients with T1e2 palpable and non-palpable breast cancer	19.7%(233/1185)	Suspicion of multifocal disease	2.81	1.30-6.06	0.70	0.69
			Preoperative MRI-scan absent	1.80	1.02-3.18		
			Positive preoperative N-stage	1.73	0.97-3.07		
			Non-palpable tumour	1.51	1.07-2.13		
			Microcalcifications on mammogram	1.37	0.95-2.00		
			Preoperative T2-stage	1.33	0.87-2.02		
			Breast density on mammogram	1.22	1.00-1.49		
			Presence of DCIS component	3.11	2.19-4.42		
			Lobular histology	2.90	1.71-4.91		
			ER positive	1.80	1.04-3.13		
			Elston III grade	1.44	0.96-2.16		
Barentsz et al. (16)	Patients with non-palpable breast cancer	12.0%(69/576)	Microcalcifications on mammogram	2.14	1.22-3.77	0.70	0.69
			Invasive tumour size	1.75	1.20-2.56		
			Presence of DCIS component on biopsy	2.61	1.41-4.82		
			Bloom and Richardson grade 2/3	1.82	1.05-3.14		
			Caudal location within breast	2.40	1.35-4.27		
Pan et al. (13)	Patients with invasive or *in situ* palpable and non-palpable breast cancer	19.4%(232/1193)	Preoperative tumor size			0.72	0.69
			2–5 cm	1.57	1.12-2.20		
			Unknown	1.50	0.93-2.42		
			Positive preoperative N stage	6.83	4.83-9.66		
			HR-positive	2.04	1.23-3.37		
			Positive HER2	1.99	1.41-2.82		
			Suspicion of multifocality	1.83	1.04-3.21		
Ellbrant et al. (18)	Patients with invasive or in situ breast cancer	17.8%(77/432)	Visible on mammography	2.33	0.73-7.60	0.80	0.75
			ILC on needle biopsy	5.59	2.71-11.50		
			DCIS on needle biopsy	4.44	2.00-9.83		
			Distance from nipple-areola complex ≥ 5cm	2.96	1.63-5.40		
			Oncoplastic surgery	2.25	1.17-4.32		
			Mammographic calcification	1.52	0,80-2.89		
Present study (2022)	Patients with invasive or *in situ* palpable and non-palpable breast cancer	18.6%(74/398)	Tumor size in ultrasound ≤2cm	2.12	1.19-3.80	0.71	0.68
			Increased blood flow	2.88	1.55-5.35		
			Microcalcifications	2.39	1.37-4.17		
			Positive cerbB-2 status	1.99	1.02-3.85		
			Grade 1/2	2.46	1.37-4.44		

To date, only two models have been constructed using the revised definition (13, 18). However, the conclusion obtained from Ellbrant’s study may be not applicable to Chinese patients because breast density and size varies from foreign to Chinese women (26). Pan’s study, implemented in China, differs from ours as their primary outcome was frozen status and included neoadjuvant chemotherapy (NAC) patients. The evidence shows that the imaging is significantly different and complex after preoperative treatment (27, 28). As a result, the model including those patients would inevitably have limitations when applied to the general population. In light of the authors’ recommendation, intraoperative frozen margin evaluation can be avoided if the positive probability is less than 10%. In contrast, a larger resection or oncoplastic surgery could be recommended if the probability of intraoperative positive margin is more than 30%. The rate of secondary operations could rise to 20-40% without interoperation assessment (3). These cutoff levels may be reasonable for both surgeons and patients. Similarly, our results indicate that intraoperative assessment can be excluded when the score is less than eight. In addition, the surgeon may attempt a wider excision or recommend plastic surgery if the score is higher than 21. Using our model, clinicians can advise patients on avoiding interoperation assessment, the likelihood of requiring interoperation, re-excision, or reoperation, allowing a more patient-centered approach. Our pragmatic and simple model is based on five preoperative factors: microcalcifications on mammography, blood flow signals on ultrasound, HER-2 status, tumor diameter, and grade. These factors partially overlap with those of other margin positivity prediction models.

One of the novel findings of our study was that tumors with increased blood flow signals are a significant predictor of margin status. Abundant blood flow is one prominent feature of malignancy (29) and can be easily measured in clinical practice. Our findings suggest that this indicator could be considered in a future model development to improve model precision. This study did not collect information about intraductal components in the puncture sample, largely because it was inaccurate. According to previous studies, the false-negative rate could be as high as 32% and 46% (30, 31). Moreover, the clinical status of the lymph nodes before surgery was not included in the model for the same reason.

Our model shows good discrimination in the modeling and validation sets with acceptable calibrations. However, external multicentric validation is needed to test its generalization. Furthermore, breast density was not considered in this study, which correlates well in some models but is not regularly reported by our institution. Multivariate analysis failed to detect any association between multiple lesions, margin status, and the possibility of improving the model’s accuracy. Since MRI is not available at many institutions, it might not be feasible to include it in the model. Hence, suspicion of multifocality was excluded from the nomogram. Another limitation of this study is the retrospective design. Further prospective studies are needed to test the clinical benefits of this model.

## Conclusion

The clinicopathological variables in our nomogram were used to predict the probability of positive margins after lumpectomy. Furthermore, we assessed the risk of bias and the model’s applicability using PROBAST (Prediction model Risk of Bias Assessment Tool) (32). Our nomogram can assist surgeons in identifying high- and low-risk patients and facilitate decision-making by surgeons and patients before BCS.

## Data Availability Statement

The raw data supporting the conclusions of this article will be made available by the authors, without undue reservation.

## Ethics Statement

The studies involving human participants were reviewed and approved by Institutional Review Board of Shanxi Bethune Hospital. The ethics committee waived the requirement of written informed consent for participation.

## Author Contributions

Conceptualization: JG, RZ, and JX. Project administration: JG. Methodology: RZ and JG. Data Curation: RZ and JX. Formal analysis: RZ. Manuscript preparation: JG and RZ. Final approval of manuscript: all authors.

## Funding

This article was sponsored by the “136 Medical Project” by Shanxi provincial government.

## Conflict of Interest

The authors declare that the research was conducted in the absence of any commercial or financial relationships that could be construed as a potential conflict of interest.

## Publisher’s Note

All claims expressed in this article are solely those of the authors and do not necessarily represent those of their affiliated organizations, or those of the publisher, the editors and the reviewers. Any product that may be evaluated in this article, or claim that may be made by its manufacturer, is not guaranteed or endorsed by the publisher.
